# Positive and Detached Reappraisal of Threatening Music in Younger and Older Adults

**DOI:** 10.3389/fnhum.2020.00216

**Published:** 2020-06-25

**Authors:** Sandrine Vieillard, Charlotte Pinabiaux, Emmanuel Bigand

**Affiliations:** ^1^Unité DysCo, Fonctionnement et Dysfonctionnement Cognitifs: Les âges de la vie, Université Paris Nanterre, Nanterre, France; ^2^Unité LEAD, Laboratoire d’Etude de l’Apprentissages et du Développement, UMR CNRS 5022, Université Bourgogne-Franche-Comté, Dijon, France

**Keywords:** age-related effects, positive reappraisal, detached reappraisal, affective outcomes, behavioral responses, physiological measures, cognitive cost

## Abstract

Past empirical studies have suggested that older adults preferentially use gaze-based mood regulation to lessen their negative experiences while watching an emotional scene. This preference for a low cognitively demanding regulatory strategy leaves open the question of whether the effortful processing of a more cognitively demanding reappraisal task is really spared from the general age-related decline. Because it does not allow perceptual attention to be redirected away from the emotional source, music provides an ideal way to address this question. The goal of our study was to examine the affective, behavioral, physiological, and cognitive outcomes of positive and detached reappraisal in response to negative musical emotion in younger and older adults. Participants first simply listened to a series of threatening musical excerpts and were then instructed to either positively reappraise or to detach themselves from the emotion elicited by music. Findings showed that, when instructed to simply listen to threatening music, older adults reported a more positive feeling associated with a smaller SCL in comparison with their younger counterparts. When implementing positive and detached reappraisal, participants showed more positive and more aroused emotional experiences, whatever the age group. We also found that the instruction to intentionally reappraise negative emotions results in a lesser cognitive cost for older adults in comparison with younger adults. Taken together, these data suggest that, compared to younger adults, older adults engage in spontaneous downregulation of negative affect and successfully implement downregulation instructions. This extends previous findings and brings compelling evidence that, even when auditory attention cannot be redirected away from the emotional source, older adults are still more effective at regulating emotions. Taking into account the age-associated decline in executive functioning, our results suggest that the working memory task could have distracted older adults from the reminiscences of the threat-evoking music, thus resulting in an emotional downregulation. Hence, even if they were instructed to implement reappraisal strategies, older adults might prefer distraction over engagement in reappraisal. This is congruent with the idea that, although getting older, people are more likely to be distracted from a negative source of emotion to maintain their well-being.

## Introduction

The most widely known model of emotion regulation (Gross, 1998) proposes several emotion-regulation modalities falling along a continuum from less (i.e., situation selection, situation modification, attentional deployment) to more cognitively demanding (i.e., cognitive reappraisal, behavioral suppression) emotion-regulation modalities. Among them, cognitive reappraisal, defined as the aim to change an emotional response by reinterpreting the meaning of the emotional event, has received increasing attention over the past decade in the field of psychological aging. Nevertheless, the studies on age-related changes in the self-reported use of cognitive reappraisal yield mixed outcomes. Although a majority reveal no significant effect of aging (Hess et al., 2010; Li et al., 2011; Tucker et al., 2012; Brummer et al., 2014), some other studies report greater preference for cognitive reappraisal over other emotion-regulation modalities for older adults compared with their younger counterparts (John and Gross, 2004; Urry and Gross, 2010; Gerolimatos and Edelstein, 2012).

Researchers operationalize cognitive reappraisal in varied ways mainly with the objective to study age-related effects on the efficiency of emotional regulation. Some make a clear distinction between two main categories of positive and detached reappraisal, which were, respectively, associated with instructions consisting of *thinking about positive aspects* or *adopting a detached and unemotional attitude* while seeing an emotional scene in order to feel less negative emotion (Shiota and Levenson, 2009; Lohani and Isaacowitz, 2014; Liang et al., 2017; Livingstone and Isaacowitz, 2018). In some other studies, the authors directly liken the notion of cognitive reappraisal to detached emotion regulation by asking participants to distance themselves from the emotional event (Winecoff et al., 2011; Tucker et al., 2012; Pedder et al., 2016). Still others do not make any distinction between positive and detached reappraisal and are based on an experimental design in which participants are just asked to *decrease* the (negative) emotion they felt (Opitz et al., 2012; Allard and Kensinger, 2017). At last, positive and detached reappraisal instructions are sometimes employed as two interchangeable options of a same and unique emotion-regulation modality (Allard and Kensinger, 2014).

Not surprisingly, these considerable variations in the operationalization of cognitive reappraisal lead to equivocal findings. The measures recorded in these previous studies (i.e., affective outcomes, facial expression, physiological reactions, and—much more rarely—eye-gaze deployment) indeed show that older adults are sometimes less (Winecoff et al., 2011; Opitz et al., 2012; Tucker et al., 2012; Allard and Kensinger, 2014; Scheibe et al., 2015; Pedder et al., 2016), sometimes equally (Allard and Kensinger, 2017; Livingstone and Isaacowitz, 2018), or sometimes better (Lohani and Isaacowitz, 2014) able to successfully implement cognitive reappraisal in comparison with their younger counterparts. Authors who show that older adults are better at regulating their emotions explain their findings within the dominant framework of socio-emotional selectivity theory (SST; Carstensen et al., 1999). This theoretical model posits that the perceived time remaining in life is a critical determinant of motivational processes in life span. While getting older, the consciousness of the limited remaining time in life leads older adults to be more motivated to maintain or enhance social and emotional well-being. Consequently, older adults preferentially process positive information over negative, resulting in a positivity effect that has been widely documented in literature on aging and emotion. Accumulated findings indicate that, when cognitive resources are experimentally distracted, the positivity effect is no longer observed, suggesting that this effect is cognitively demanding processing (e.g., Knight et al., 2007). Basically, the SST model postulates that the positivity effect operates as an emotion-regulation goal (but see Isaacowitz and Blanchard-Fields, 2012, for a critical view of this hypothetical link between the positivity effect and the outcome of positive affective experience). According to this view, intentional emotion regulation is, by essence, a cognitively demanding task. Alternative but most confidential theoretical frameworks of the dynamic integration theory (DIT; Labouvie-Vief, 2008) attempt to offer a different explanation to interpret the improvement in emotional regulation with aging. As the SST model, DIT assumes that emotion regulation is cognitively costly. Focusing on the effect of cognitive decline on emotional processing, the DIT model postulates that older adults may still be able to regulate their emotion only when facing emotional stimuli of low intensity. When facing highly negative emotions, older adults tend to automatically distance from them (e.g., Brady et al., 2018), then abolishing the cognitive cost elicited by the elaborate processing of negative events considered as cognitively much more complex (Labouvie-Vief, 2008). SST assumes that the more cognitive resources older adults have, the better they are able to reappraise the emotional significance of the event, whereas DIT argues that the fewer cognitive resources older adults have, the better they are able to downregulate negative emotion, not through an emotional reappraisal *per se* (which would be too resource demanding), but through a mechanism allowing them to disengage from the source of emotion. According to the DIT model, such a mechanism would take place in an automated way precisely because the older adults’ cognitive resources are limited.

Regarding the different types of cognitive reappraisal modalities, several studies suggest that positive and detached reappraisals are not equally affected by age. For instance, Shiota and Levenson (2009) demonstrate that, even if older adults report greater success than younger adults at implementing both types of reappraisal when viewing sad and disgusting films, the age-related effect on affective outcomes and physiological reactivity varies as a function of strategy. Older adults use positive reappraisal more successfully than younger adults, whereas younger adults use detached reappraisal more successfully. Corroborating these previous findings, Lohani and Isaacowitz (2014) show that older adults are more successful than younger adults at implementing positive reappraisal in response to sadness-eliciting film clips. This is shown by the fact that, relative to the no-regulation condition, older adults report a decreased negative mood with no heightened physiological activity, and younger adults do not experience a decrease in negative mood, but an increase in physiological response. Going one step further, Liang et al. (2017) conducted a study on a single sample of older adults providing evidence that, in comparison with positive reappraisal, detached reappraisal relies more heavily on cognitive control, especially on mental set shifting used to capture mental flexibility. These results are interpreted as corroborating the idea that, given that positive reappraisal necessitates maintaining attention on emotion rather than inhibiting it, it is finally not surprising that such emotion regulation is less cognitively demanding compared to detached reappraisal. These data are also in line with the hypothesis that positive reappraisal is effective in everyday life (Guiliani and Gross, 2009; McRae et al., 2012) notably because of its benefits on mental health in the context of physical illness or stress (Nowlan et al., 2014, 2016). Although the studies above suggest the existence of a differential impact of positive and detached reappraisal depending on age, other recent findings indicate that both age groups show mood improvement when using cognitive appraisal whatever the positive or detached modality (Allard and Kensinger, 2017; Livingstone and Isaacowitz, 2018). A recent meta-analysis on age-related differences in ability to implement emotion-regulation instruction, including previously cited studies and also unpublished sources of data, concludes that both young and older adults better regulate the behavioral indicators of emotion when using detached reappraisal relative to positive reappraisal (Brady et al., 2018). In view of such discrepant findings, further investigative efforts are needed.

An important aspect of the previously cited works is that they were conducted using emotional visual scenes (film clips or pictures). Most of them often neglect the possibility that less-demanding modalities of emotion regulation, such as attention deployment using gaze direction, might help to achieve an emotion-control goal. This is a question of importance because it has been shown that older adults deploy their visual attention away from negative stimuli (Isaacowitz et al., 2006). Moreover, when controlling gaze direction, older people are less successful than younger adults at regulating unpleasant emotion elicited by a visual scene (Opitz et al., 2012). More recent findings indicate that, when getting older, distraction (avoidance by gaze redirection) is less cognitively effortful than reinterpreting negative information through a positive reappraisal instruction (Martins et al., 2018). It is then reasonable to think that the potential preferential use of attentional deployment could partly explain successful emotion regulation in older adults. This assumption is consistent with empirical evidence that, when alternative modalities are easily reachable and just as efficient, older adults prefer to use less cognitively demanding modalities of emotion regulation (Scheibe et al., 2015). This raises the question of whether, with no possibility of recourse to attentional deployment, the effortful processing of some cognitive reappraisal contexts is really spared from the general age-related decline. One way to address this issue is to use emotional stimuli that do not allow participants to reallocate perceptual attention away from the emotional source. In this respect, music represents an ideal mean. Music has also been shown to be sensitive to the age-related positivity effect (Vieillard and Gilet, 2013; Vieillard and Bigand, 2014). Using musical material ensures that participants remain engaged in the auditory processing of emotional material, but this is not to say that, once the emotion is processed, the participant could not implement an emotion-regulation strategy.

Another important aspect of the works described above is that they mainly focus on affective, behavioral, and physiological consequences of reappraisal, failing to address the impact of positive and detached reappraisal on cognitive processing. Previous studies addressing the cognitive cost of reappraisal in younger adults have yielded mixed conclusions. Some authors suggest that the use of cognitive reappraisal, in its detached version, may lead to lesser cognitive costs compared with other emotion-regulation modalities, such as expressive suppression (Richards and Gross, 2000; Richards et al., 2003; Gross and Thompson, 2007). For instance, Richards and Gross (2000) find that participants who receive an instruction to detach from their feelings before watching pictures of injured people show better memory for the picture details compared to those who are told to use expressive suppression or no regulation. Richards et al. (2003) show that the memory for conversion utterances is increased when people are told to positively reappraise their emotional inner states during a naturalistic conflictual discussion compared to when they are told to suppress the expression of their feelings. Some researchers question such less cognitive cost, arguing that the successful use of cognitive reappraisal, whether positive or detached, requires active reinterpretation of the meaning and significance of emotional stimuli and, thus, involves demanding processes, such as working memory, flexibility, or inhibition, especially in emotionally intense situations (Hofmann et al., 2012; Ortner et al., 2016). Hence, it has been shown that detached reappraisal is associated with decreased performance on reaction-time tasks and decreased self-control resources when used in high-intensity negative situations (Sheppes and Meiran, 2008; Ortner et al., 2016). Detached reappraisal of positive pictures is also shown to cause a decrease in subsequent memory recognition (Ortner and de Koning, 2013). On a subjective level, positive reappraisal seems to be more difficult to implement than acceptance as indicated by the greater perceived cognitive cost of positive reappraisal reported by young adults (Troy et al., 2018). To date, cognitive consequences of detached and positive reappraisals has never been compared in a within-subject design experiment, leaving open the question as to whether one of them could be less costly than the other in younger adults. Regarding the effect of age, to our knowledge, only one study has attempted to test whether cognitive reappraisal was synonymous with higher cognitive cost in advancing age. Scheibe and Blanchard-Fields (2009) investigate the age-related effect on cognitive consequences of intentional downregulation of disgust induced by film clips. To this end, they asked younger and older participants to implement a positive reappraisal consisting of turning negative feelings potentially elicited by the emotional stimulus into positive ones. Measuring the working memory performances as a cognitive load index of the emotion regulation activity with a *N*-back task, the authors find that in comparison with their younger counterparts, older adults show better working memory performances when implementing positive reappraisal after emotional induction. Given the cognitive control *a priori* required by the execution of positive reappraisal, such findings may sound counterintuitive in particular with regard to the age-related decline in executive functioning. In an attempt to provide a plausible account, Scheibe and Blanchard-Fields (2009) reason in terms of long-term practice in regulating emotion while getting older, speculating that such practice might render the emotion regulation activity less costly. Another explanation in terms of distraction is advanced by authors with the idea that the working memory task itself could be operated in an emotion-regulation way, distracting older adults from their memories of the negative emotion elicited by film clips. Nevertheless, authors do not consider the possibility that the emotion regulation has been less cognitively costly in older adults precisely because they preferentially avert their eyes from disgusting film clips. To determine which of these two hypotheses best accounts for Scheibe and Blanchard-Fields (2009) results, we need to replicate and extend their findings in a context in which participants cannot reallocate perceptual attention away from the emotional source. We conduct such a study to investigate the effect of age on both positive and detached reappraisal with the hope to provide a thinner understanding on emotion-regulation consequences in aging. We also addressed this issue with an attempt to determine which of the theoretical frameworks, SST or DIT, offers a better account for empirical findings.

In the current study, we examine the affective, behavioral, physiological, and cognitive outcomes of positive and detached reappraisal in response to negative musical emotion in both younger and older adults. Participants were first asked to simply listen to a series of threatening musical excerpts and then were given the instruction to either positively reappraise or to detach themselves from the emotion elicited by music. The participants’ affective ratings (Likert scale), facial expressions (facial EMG), and physiological state (SCL, HR) were recorded as a spontaneous control condition during the simply listening phase as well as under the instruction of emotion regulation. The simply listening condition allowed us to examine how the affective state of older adults spontaneously changes when listening to threatening music. In line with previous findings (Vieillard and Gilet, 2013; Vieillard and Bigand, 2014), we expected that the affective ratings of the threatening musical excerpts would be less negative and intense for older adults than for younger ones. This may be associated with age-related changes in facial expressions and physiological state reflecting the tendency of older adults to reduce the processing of negative emotion. Regarding the cognitive reappraisal, the scarce and contradictory results do not facilitate the formulation of specific hypotheses on affective outcomes, expressive responses, and physiological reactions. However, if we rely on the dominant framework of the SST, it would be expected that, whatever the kind of cognitive reappraisal, older adults would report more positive affective outcomes and expressive responses than their younger counterparts. Because the SST postulates that reappraisal is cognitively costly, it is reasonable to postulate that this cost would be reflected in more physiological reaction. The DIT framework predicts similar findings on the condition that threatening musical excerpts are experienced as having relatively low intensity. If threatening musical excerpts are experienced as having high intensity, the DIT conjectures that, compared to younger adults, older adults would be less effective at implementing emotion-regulation instructions, whatever the kind of reappraisal.

We also wanted to test the robustness of what appears to be better emotional control with age. To extend previous findings to a different cognitive task, we use a memory span task adapted from Schmeichel (2007) work. Our goal was to measure cognitive performances of participants just after implementing the simply listening instruction as well as reappraisal (positive, detached) instructions. As postulated by the SST, if we consider that the spontaneous modulation of negative affects operated by older adults is resource demanding, it should lessen their subsequent working memory performance more than for younger adults, whatever the spontaneous or intentional condition. On the other hand, as stated by the DIT, if older adults spontaneously downregulate their negative feelings in an automated way when faced with not too emotionally intense stimuli, their subsequent working memory performance should not be affected by their spontaneous emotion regulation or by their intentional emotion regulation, contrary to younger adults. Moreover, based on Scheibe and Blanchard-Fields (2009) findings showing that reappraisal is less effortful as people grow older, we hypothesize that reappraisal in our study would be less cognitively demanding in older adults in comparison with younger adults. A plausible explanation is that focusing on the working memory task may distract participants from the emotions elicited during the emotion regulation (Scheibe and Blanchard-Fields, 2009). Because distraction has been demonstrated to be an efficient emotion-relation modality in aging (Martins et al., 2018), we can sketch out a general prediction that, whatever its positive or detached type, the cognitive reappraisal would be less costly in older than in younger adults. Given that, in our experiment, participants cannot avoid listening to musical excerpts, a replication of Scheibe and Blanchard-Fields (2009) results would mean that, in line with previous works (e.g., Vieillard and Bigand, 2014), older adults have higher propensity to disengage from negative stimuli, in particular when they are helped by a source of distraction (working memory task). This would be in line with DIT (Labouvie-Vief, 2008) and agree with the hypothesis that older adults become more efficient at implementing intentional emotion regulation (not based on real reappraisal but on distraction). In the case in which we do not replicate Scheibe and Blanchard-Fields (2009) results, an alternative explanation, in line with the SST, would be that the implementation of a cognitive reappraisal strategy, whether positive or detached, may importantly lessen the working memory performances of older adults in comparison with their younger counterparts.

## Materials and Methods

### Participants

Forty-six non-musician younger adults from the University of Franche-Comté, France, and 37 non-musician older adults recruited through senior social programs in Besançon, France, participated in the experiment. We ensured that they had no neurological or psychiatric antecedent and reported normal or corrected visual acuity. Due to the affective aspect of the experiment, we excluded 11 younger and three older adults reporting high depressive symptoms (BDI-II score higher than 30) and/or high levels of anxiety (state and trait STAI-Y standard scores higher than 55) from the analyses. The final sample consists of 35 younger adults ranging from 18 to 27 years (*M* = 21, *SD* = 2.23; 60% females) and 34 older adults ranging from 60 to 79 years (*M* = 66, *SD* = 4.80; 62% females).

As illustrated in Table 1, older adults did not report statistically different levels of education or self-reported health, but younger adults reported spending more time on music listening per week (*M* = 10.97 h, *SD* = 10.99) than older adults (*M* = 7.55 h, *SD* = 11.34; *p* = 0.036). For each participant, potential hearing loss was examined using a professional audiometer. As expected, the hearing level (dB) varied as a function of age group. The examination of cognitive functioning showed that younger adults have higher performance on the Victoria Stroop index (Bayard et al., 2011) than older adults, but no age-related effect was found on the letter–digit sequencing test (WAIS-III, Wechsler, 2000). The investigation of affective functioning indicated that older and younger adults did not significantly differ on depression (BDI-II, Beck et al., 1996), state anxiety (STAI- Y; Spielberger et al., 1983), trait anxiety (STAI-Y; Spielberger et al., 1983), PANAS positive affects (Watson et al., 1988), and reappraisal subscale of ERQ (Christophe et al., 2009) measures. However, there was a statistically significant main effect of age on PANAS negative affects (Watson et al., 1988). Finally, the examination of personality traits (measured by the French validation of NEO-P-IR by Plaisant et al., 2010) showed age-related differences on the mean scores of extraversion. No other significant difference was found.

**TABLE 1:** Sample characteristics.

	**Younger adults (*n* = 35)**	**Older adults (*n* = 34)**	**Shapiro–Wilk Test**	**Levene’s homogeneity test**	**Age group difference**
	**Mean**	**Mean**	***p*-value**	***p*-value**	***Corrected p* value^*a*,*b*^**
**Demographic characteristics**					
Age (year)	21 (2.22)	66 (4.81)	–	–	0.000^(b)^
Education (year)^(c)^	13.14 (1.55)	13.88 (2.01)	0.00	0.13	0.205^(b)^
Sex (% female)	60	62	–	–	–
Music listening per week (hours)	10.97 (10.99)	7.55 (11.34)	0.00	0.47	0.036^(b)^
Self reported health (max. 5)	4.43 (0.65)	4.32 (0.58)	0.00	0.28	0.446^(b)^
Hearing level (dB)					
500 Hz	8.43 (6.10)	15.51 (5.53)	0.00	0.62	0.000^(b)^
1000 Hz	6.86 (7.73)	15.59 (7.39)	0.00	0.48	0.000^(b)^
2000 Hz	3.07 (6.81)	18.90 (12.93)	0.00	0.00	0.000^(b)^
4000 Hz	0.57 (7.45)	30.29 (18.43)	0.00	0.00	0.000^(b)^
8000 Hz	6.86 (10.73)	47.43 (21.13)	0.00	0.00	0.000^(b)^
**Cognitive Scores**					
Inhibition: Victoria Stroop (IF)	1.77 (0.36)	2.04 (0.32)	0.59	0.31	0.002^(a)^
Working Memory: Digit Span (max. 30)	11.14 (2.85)	10.15 (1.94)	0.00	0.08	0.171^(b)^
MMSE	–	29.76 (0.50)	–	–	–
**Affectives Scores**					
PANAS positive affects (max. 50)	32.83 (5.88)	34.68 (4.72)	0.02	0.44	0.296^(b)^
PANAS negative affects (max. 50)	18.97 (6.93)	16.15 (5.58)	0.00	0.79	0.032^(b)^
BDI-II (max. 63)	7.69 (5.26)	6.09 (5.36)	0.00	0.72	0.181^(b)^
STAI-Y trait (max. 80)	38.14 (6.54)	35.09 (6.51)	0.28	0.72	0.283^(a)^
STAI-Y state (max. 80)	29.23 (5.17)	27.79 (5.84)	0.06	0.64	0.086^(a)^
**Emotion Regulation Questionnaire Scores**					
ERQ Suppression Score (max. 28)	15.83 (5.43)	13.68 (4.87)	0.15	0.66	0.127^(a)^
ERQ Regulation Score (max. 42)	27.74 (5.83)	29.53 (6.06)	0.10	0.63	0.248^(a)^
**NEO PIR Scores**					
Neuroticism (max. 192)	89.83 (22.36)	83.12 (16.23)	0.03	0.04	0.000^(b)^
Extraversion (max. 192)	114.20 (16.94)	103.30 (12.13)	0.53	0.07	0.006^(a)^
Openess to experience (max. 192)	123.20 (18.45)	117.53 (12.41)	0.81	0.03	0.000^(b)^
Consciousness (max. 192)	115.09 (21.45)	121.41 (13.02)	0.04	0.02	0.000^(b)^
Agreeableness (max. 192)	121.54 (24.26)	131.03 (14.40)	0.00	0.01	0.000^(b)^

*Standard deviations are listed in parentheses. (a) Student’s *t* test, *p* values are corrected with the Hochberg procedure for controlling the family-wise error rate; (b) Mann–Whitney *U* test, *p* values are corrected with the Hochberg procedure for controlling the family-wise error rate; (c) Number of years of education has been calculated from 6 years old (age at which school is compulsory in France).*

### Material

Forty threatening musical excerpts taken from a Platel et al. (unpublished) database of film soundtracks and three peaceful musical excerpts taken from the database of Bigand et al. (2005) were used in this experiment (Supplementary Material). Among the 40 threatening excerpts, two of them were devoted to a training phase, six were allocated to a condition in which the instruction was to spontaneously respond to music, and six were allocated to a condition in which the instruction was to implement an emotion-regulation modality of reappraisal. Two peaceful musical excerpts were added. One was used as a familiarization phase with rating scales, and the other was used as a debriefing phase. All these musical stimuli were extracted from the classic (for peaceful music) and modern repertoires (for threatening music), excluding songs. Additional musical material was used as a baseline condition and was especially created for this study in order to test whether affective, behavioral, and physiological responses to such stimuli varied as a function of age groups. These control musical stimuli consisted of four auditory stimuli, including a tuning orchestra or playing scales in cello or piano. All the musical stimuli have a duration of 20 s.

### Procedure

The experiment was divided into two sessions separated by an interval of about 2 weeks at the university of Franche-Comté. In the first session, participants were instructed to sign a consent form according to the declaration of Helsinki. They filled out a demographic questionnaire, including information about their age, education level, self-reported health, visual acuity, and medical history and were then presented with a set of cognitive and affective tests. The first session lasted about 1 h.

**FIGURE 1:**
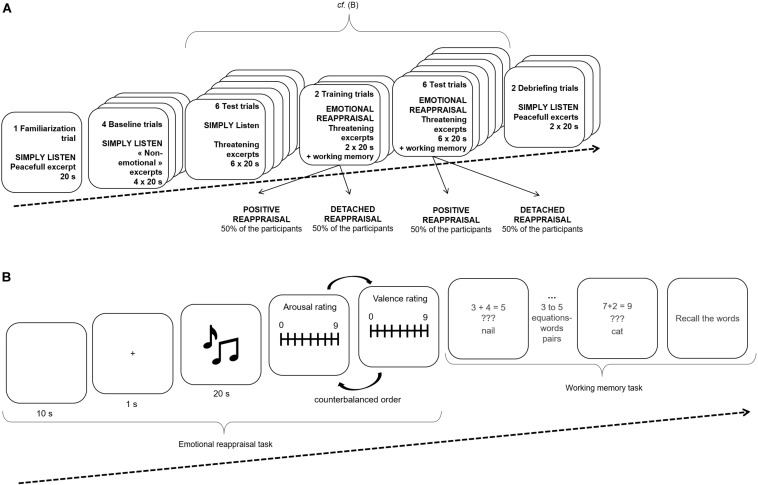
General procedure **(A)** and trial shematic **(B)** for emotion regulation task.

In the second session, participants were tested individually in a quiet room at stable ambient temperature (Figure 1A). Once the participants were installed with the physiological system, they were asked to listen to two threatening musical excerpts, one by one, in a set of training trials. These later were used to allow each participant to adjust the sound loudness so that it was judged to be as comfortable as possible. Immediately after, one peaceful excerpt was presented with the aim to familiarize each participant with rating scales designed to evaluate the intensity of the emotional experience (“The emotion I feel is” from 0 “weak” to 9 “strong”) and the hedonic valence of the emotional experience (“The emotion I feel is” from 0 “negative” to 9 “positive”). The order of presentation of the rating scales was counterbalanced across participants.

For each participant, the emotion-regulation task always began with a baseline condition followed by the simply listening condition, which was, in turn, followed by the reappraisal condition. Under the reappraisal condition, half of the participants were randomly allocated to a detached reappraisal condition, and the other half were randomly allocated to a positive reappraisal condition. As illustrated in the appendix, the assignment of the 12 threatening excerpts was controlled so that, when the first half was allocated to a simply listening condition, the other half was allocated to a reappraisal condition (either detached or positive reappraisal) and vice versa. In each emotion-regulation condition, the order of the presentation of the musical excerpts was randomized. The experiment ended with a debriefing block of two peaceful musical excerpts for which participants were asked to apply a simply listening instruction.

In the baseline condition, the four auditory excerpts were presented with the following instruction: “You will listen to auditory excerpts. Be careful because, after each excerpt, you will be asked to evaluate what you thought and felt during the listening.” The simply listening condition, including a block of six threatening musical excerpts, was presented with the following instruction: “You will listen to musical excerpts, which can elicit in you some feelings. We ask you to listen to them carefully and to feel your emotion as you want. After each musical excerpt, you will have to evaluate what you have felt during listening.” The reappraisal emotion-regulation condition was divided into two distinct instructions: a positive and a detached reappraisal. The positive reappraisal instruction was: “You will listen to musical excerpts conveying a negative feeling. While listening to the music, we ask you to reconsider what the music conveys in such a way that you will focus on its positive aspect. To this end, please try to think that this music has been composed to comic purposes in order to feel the least negative emotion as possible.” The detached reappraisal instruction was: “You will listen to musical excerpts conveying a negative feeling. While listening to the music, we ask you to detach yourself from what the music conveys. To this end, please try to think about other things than what you are listening to in order to feel the least negative emotion as possible.” After presenting the two threatening musical excerpts devoted to the training phase for the reappraisal instruction implementation and before presenting the six threatening stimuli devoted to the testing part (i.e., simply listening and cognitive reappraisal), a set of instructions related to a working memory span task (Schmeichel, 2007) was displayed. In this task, participants had to judge the correctness of math equations while encoding target words for a subsequent recall. For instance, participants saw (“3 + 4 = 5”) and had to indicate “Yes” or “No” as to whether the given answer was correct. Then the participants read a target word aloud (e.g., nail) for later recall. One target word was presented after each equation. The participants saw three, four, or five equation–word pairs before being prompted to recall the target words in the set. They did not know in advance how many words a set would include. The working memory span task included 12 sets totaling 48 equation–word pairs in all, counterbalanced across participants. The experiment was designed such that, after each of the six simply listening trials and the six reappraisal trials, the participants were presented with one trial of a memory span task. Equation–word pairs were displayed on a computer screen and participants controlled their display with their responses.

In this experiment, one trial always began with the emotion-regulation instructions after the participants indicated they were ready (Figure 1B). A 10-s blank screen was displayed while the physiological baseline was recorded, followed by a fixation cross of 1 s. Immediately after, the musical excerpt was delivered for 20 s. At the end of the musical excerpt’s presentation, the participants evaluated it on rating scales and performed one trial from the memory span task. For each of this set of events, the experimenter carefully examined the level of cutaneous conductance and did not start the next trial until it had stabilized, ensuring that this measure returned to baseline. Once the tasks were completed, the physiological system was removed, and the participant was then debriefed. The second session lasted about 1 h.

### Data Acquisition and Transformation

Physiological responses were monitored throughout the experiment using an MP150 Biopac system (Biopac Systems, Inc., Goleta, CA) at a sampling rate of 500 Hz and were processed using AcqKnowledge software. The facial expressivity of the participants was assessed through their zygomaticus electromyographical activity (facial EMG, μ volts) with two 4-mm shielded electrodes located on the left zygomaticus muscle (see recommendations of Tassinary et al., 2007). The signal was rectified using the root mean square function of the software and then smoothed using a 50-Hz band stop filter. The EMG score was calculated by subtracting the EMG signal recorded for the 1-s duration before the onset of the musical excerpt from the EMG signal (area under the curve per second) recorded for the 20 s of musical excerpt presentation. Skin conductance level (SCL, μ Siemens) was recorded on the left index and little fingers with two electrodes filled with isotonic gel. The reported SCL score was calculated by subtracting the SCL signal recorded at the onset of the musical excerpt from the SCL recorded from the third second to the end of the musical excerpt presentation. Electrocardiogram (heart rate in beats per minute – HR in bpm) activity was recorded with three 8-mm electrodes located at the left wrist (+), right wrist (-), and left ankle (ground). The signal was smoothed using a 50-Hz band stop filter. The reported HR score was calculated by subtracting the HR signal recorded during the 1-s before the onset of the musical excerpt from the HR signal recorded during the 20-s of musical excerpt presentation. The distribution of each variable was examined to identify possible remaining outliers (mean ± 3 *SD*). Based on this criterion, about 3.4% of all measurements were excluded from the analyses.

### Data Analyses

The normality and homogeneity of variances were tested using Shapiro Wilk and Levene’s tests before the statistical analyses were applied. Because these conditions were not met, the different variables were analyzed with non-parametric statistics using Mann–Whitney *U* tests for between-groups comparisons. For each affective (arousal and valence judgment), expressive (facial EMG), physiological (SCL and HR), and cognitive (mean ratio at the working memory task) measure, we conducted the analyses on the efficiency of the reappraisal strategies (positive vs. detached) with a differential score obtained by subtracting the measure obtained for the cognitive reappraisal condition from the measure obtained in the simply listening condition. Positive scores corresponded to relatively higher value of the measure under the reappraisal instructions compared to the simply listening condition. On the contrary, negative scores corresponded to higher values of the measure in the simply listening condition compared to the reappraisal instruction. The efficiency of both types of reappraisal was then tested for each age group separately (one-sample *t* test, comparison of the differential score with μ = 0) and compared between age groups (Mann–Whitney *U* tests). In the end, the efficiency of the two types of reappraisal (positive vs. detached) was compared within each age group (Mann–Whitney *U* tests). To control for the equivalence between each age group (younger vs. older) through reappraisal conditions (positive vs. detached) two sets of Mann–Whitney *U* tests were conducted. Because multiple comparisons are conducted, we applied the Hochberg procedure for controlling the family-wise error rate to correct the *p* values. To investigate the consistency between subjective ratings and cognitive cost of emotion-regulation instructions, correlation analyses were calculated for each experimental condition separately for younger and older adults. We also examined how the affective and the cognitive functioning relate to emotion regulation across age groups. Every time that age-related differences were found, we computed a set of correlation analyses between sample characteristics and the differential scores of the affective, behavioral, physiological, and cognitive measures for each experimental condition, separately for younger and older adults.

## Results

### Age Equivalence in Baseline Reactivity Levels (Control Musical Excerpts)

As shown in Table 2, during the baseline condition, the older adults reported a more negative emotional experience than the younger adults (*p* = 0.036) together with an increased HR compared to their younger counterparts (*p* = 0.045). However, both age groups reported a globally negative (i.e., <4,5/9) emotional experience when listening to the so-called control musical excerpts. No other statistically significant difference between groups was found.

**TABLE 2:** Age equivalence in baseline condition (control musical stimuli).

	**Younger adults (*n* = 35)**	**Older adults (*n* = 34)**	**Age comparisons**
	**Mean**	**Mean**	**Corrected *p*-value**
**Affective responses**			
Arousal rating (max. 9)	3.250 (1.463)	3.956 (2.341)	0.478
Valence rating (max. 9)	3.979 (1.085)	3.125 (1.790)	0.036
**Expressive responses**			
Facial EMG Zygomaticus Major (Area under the curve, mV*sec)	0.002 (0.008)	0.0002 (0.0008)	0.593
Facial EMG Corrugator Supercilii (Area under the curve, mV*sec)	0.0009 (0.001)	0.0007 (0.0011)	0.474
**Physiological responses**			
SCL’s magnitude (μS)	0.0250 (0.0630)	0.0120 (0.0330)	0.108
HR (bpm)	−0.0493 (3.586)	0.6786 (3.715)	0.045

*Standard deviations are listed in parentheses. Age comparisons were assessed by MannWhitney *U* test. *p* values are corrected with the Hochberg procedure for controlling the family-wise error rate.*

### Age-Related Effects on Spontaneous Responses to Threatening Musical Excerpts

As shown in Table 3, our results indicate that, in comparison with their younger counterparts, the older adults has a more positive emotional experience (*p* = 0.038) and showed a smaller SCL (*p* = 0.007). No other statistically significant difference between age groups was observed.

**TABLE 3:** Spontaneous responses to threatening musical excerpts in younger and older adults (simply listen condition).

	**Younger adults (*n* = 35)**	**Older adults (*n* = 34)**	**Age comparisons**
	**Mean**	**Mean**	**Corrected *p*-value**
**Affective responses**			
Arousal rating (max. 9)	5.295 (1.280)	5.476 (1.646)	0.671
Valence rating (max. 9)	4.024 (1.176)	4.843 (1.651)	0.038
**Expressive responses**			
Facial EMG Zygomaticus Major (Area under the curve, mV*sec)	0.0016 (0.0052)	0.0003 (0.0006)	0.673
Facial EMG Corrugator Supercilii (Area under the curve, mV*sec)	0.0006 (0.0012)	0.0005 (0.0006)	0.947
**Physiological responses**			
SCL’s magnitude (μS)	0.1663 (0.1342)	0.0815 (0.1187)	0.007
HR (bpm)	−0.493 (3.586)	0.371 (3.913)	0.671
**Working memory**			
Mean ratio	0.7400 (0.1500)	0.7500 (0.1100)	0.671

*Standard deviations are listed in parentheses. Age comparisons were assessed by Mann–Whitney *U* test. *p* values are corrected with the Hochberg procedure for controlling the family-wise error rate.*

### Age-Related Effects on Cognitive Reappraisal

#### Positive Reappraisal

As shown in Table 4, the set of one-sample *t* tests (μ = 0) indicates that, when they were asked to positively reappraise threatening musical excerpts, both younger (*p* = 0.007) and older (*p* = 0.033) adults reported a significantly more positive emotional experience than when they were asked to simply listen to it. No statistically significant effect of positive reappraisal instruction was found on the reported intensity of the emotional experience, whatever the age group. Similarly, neither expressive nor physiological responses (SCL, HR) varied as a function of the instruction to positively reappraise musical excerpts, whether in younger or older adults. Concerning the cognitive cost of positive reappraisal, when they were asked to positively reappraise the musical excerpts, the older adults showed a significant gain in their working memory performances in comparison with the simply listening condition (*p* = 0.007). This is not the case in the younger adults, who showed no beneficial effect when implementing positive reappraisal. The age group comparisons confirm that the gain in working memory performances observed in the positive reappraisal condition is specific of older adults (*p* = *0.042*). No other statistically significant difference between age groups was found.

**TABLE 4:** Positive reappraisal’s efficiency (differential score: reappraisal condition minus simple listening condition) in younger and older adults.

	**Younger adults (*n* = 18)**		**Older adults (*n* = 17)**		**Age comparisons between differential scores**
	**Mean**	**Statistical difference from 0 (corrected *p*-value)**	**Mean**	**Statistical difference from 0 (corrected *p*-value)**	**Corrected *p*-value**
**Affective responses**					
Arousal rating (max. 9)	−0.3796 (1.2895)	0.534	−0.2059 (1.5916)	0.701	0.792
Valence rating (max. 9)	1.2130 (1.3368)	0.007	0.8039 (1.1935)	0.033	0.510
**Expressive responses**					
Facial EMG Zygomaticus Major^a^	−0.0007 (0.0015)	0.228	0.0001 (0.0015)	0.793	0.273
Facial EMG Corrugator Supercilii^a^	0.0000 (0.0012)	0.883	−0.0001 (0.0005)	0.430	0.711
**Physiological responses**					
SCL’s magnitude (μS)	0.0077 (0.0647)	0.725	−0.0154 (0.0231)	0.033	0.299
HR (bpm)	0.8733 (4.7497)	0.725	−1.0350 (3.5762)	0.430	0.273
**Working memory**					
Mean ratio	0.0107 (0.0879)	0.725	0.0869 (0.0917)	0.007	0.042

Standard deviations are listed in parentheses. Age comparisons between differential scores were assessed by Mann–Whitney *U* test, whereas statistical differences from 0 were assessed by Student’s *t* test. ^*a*^Area under the curve, mV* sec. *p* values are corrected with the Hochberg procedure for controlling the family-wise error rate.

#### Detached Reappraisal

As illustrated in Table 5, the set of one-sample *t* tests (μ = 0) indicates that, when they were asked to detach from the emotion elicited by threatening musical excerpts, older (*p* = 0.038) but not younger (*p* = 0.133) adults reported a significantly less negatively valenced experience than when they were in the simply listening condition. No difference was observed for arousing ratings. Neither the expressive nor the physiological responses (SCL, HR) varied as a function of the instruction to use detached reappraisal, whether in younger or older adults. Once again, our findings indicate that, when they were told to detach from the emotion elicited by the threatening musical excerpts, the older adults showed a statistically significant gain in their working memory performances in comparison with the simply listening condition (*p* = 0.038). This is not the case in the younger adults. However, this is not associated with a significant age group comparison effect (*p* = 0.493). No other statistically significant difference between age groups was found.

**TABLE 5:** Detached reappraisal’s efficiency (differential score: reappraisal condition minus simple listening condition) in younger and older adults.

	**Younger Adults (*n* = 17)**		**Older Adults (*n* = 17)**		**Age comparisons between differential scores**
	**Mean**	**Statistical difference from 0 (corrected *p*-value)**	**Mean**	**Statistical difference from 0 (corrected *p*-value)**	**Corrected *p*-value**
**Affective responses**					
Arousal rating (max. 9)	−1.1476 (1.8199)	0.133	−0.1.3235 (1.8648)	0.038	1.00
Valence rating (max. 9)	0.3529 (1.0815)	0.345	−0.7843 (1.3081)	0.058	0.154
**Expressive responses**					
Facial EMG Zygomaticus Major^a^	0.0020 (0.0069)	0.346	−0.0000 (0.0006)	0.930	0.369
Facial EMG Corrugator Supercilii^a^	−0.0002 (0.0023)	0.772	0.0002 (0.0005)	0.296	0.883
**Physiological responses**					
SCL’s magnitude (μS)	−0.0216 (0.0474)	0.184	−0.0005 (0.0239)	0.930	0.369
HR (bpm)	−2.3197 (4.9224)	0.184	−0.1307 (4.5720)	0.930	0.399
**Working memory**					
Mean ratio	0.0241 (0.1143)	0.466	0.0600 (0.0865)	0.038	0.493

*Standard deviations are listed in parentheses. Age comparisons between differential scores were assessed by Mann–Whitney *U* test, whereas statistical differences from 0 were assessed by Student’s *t* test. ^a^Area under the curve, mV^∗^sec. *p* values are corrected with the Hochberg procedure for controlling the family-wise error rate.*

#### Positive and Detached Reappraisal Together

When analyzed together, the impact of both positive and detached reappraisal yields supplementary results as shown in Table 6. First, the set of one-sample *t* tests (μ = 0) indicates that the instruction to reappraise the musical excerpts led to a less arousing emotional experience for younger (*p* = 0.032) but not older (*p* = 0.067) adults. Younger adults also reported a more positive emotional experience when they were asked to reappraise than when they were not (*p* = 0.007). Concerning the cognitive cost of reappraisal, we show again that older adults benefit from the instruction to reappraise their emotional experience in a way that their working memory performances improve in comparison with the simply listen condition (*p* = 0.001). No effect of the instructions on the behavioral or physiologic measures was found. We found no significant age group comparisons.

**TABLE 6:** Cognitive (positive and detached) reappraisal’s efficiency (differential score: reappraisal condition minus simple listening condition) in younger and older adults.

	**Younger adults (*n* = 35)**		**Older adults (*n* = 34)**		**Age comparisons between differential scores**
	**Mean**	**Statistical difference from 0 (corrected *p*-value)**	**Mean**	**Statistical difference from 0 (corrected *p*-value)**	**Corrected *p*-value**
**Affective responses**					
Arousal rating (max. 9)	−0.7524 (1.5942)	0.032	−0.7647 (1.7986)	0.067	0.952
Valence rating (max. 9)	0.7952 (1.2783)	0.007	0.0098 (1.4731)	0.969	0.140
**Expressive responses**					
Facial EMG Zygomaticus Major^a^	0.0006 (0.0050)	0.595	0.0000 (0.0012)	0.969	0.952
Facial EMG Corrugator Supercilii^a^	−0.0001 (0.0017)	0.852	0.0000 (0.0005)	0.969	0.952
**Physiological responses**					
SCL’s Magnitude (μS)	−0.0065 (0.0580)	0.595	−0.0080 (0.0243)	0.152	0.952
HR (bpm)	−0.6776 (5.0303)	0.595	−0.5828 (4.0677)	0.717	0.952
**Working memory**					
Mean ratio	0.0172 (0.1003)	0.595	0.0735 (0.0888)	0.001	0.091

Standard deviations are listed in parentheses. Age comparisons between differential scores were assessed by Mann–Whitney *U* test, whereas statistical differences from 0 were assessed by Student’s *t* test. ^*a*^Area under the curve, mV* sec. *p* values are corrected with the Hochberg procedure for controlling the family-wise error rate.

Table 7 shows the comparison between the positive and the detached reappraisal conditions for affective, expressive, physiological, and cognitive outcomes in the younger and older adults, respectively. No statistically significant difference between the two groups of younger adults, respectively, assigned to positive and detached reappraisal was found regardless of the measure considered. Regarding the older adults, one statistically significant difference between the two groups, respectively, assigned to positive and detached reappraisal was found for valence rating (*p* = 0.035): The older adults who were asked to positively reappraise the musical excerpts reported significantly more positive ratings of their emotional experience than those who were asked to implement the detached modality.

**TABLE 7:** Comparison between positive and detached reappraisal’s efficiency in younger and older adults.

	**Younger adults (*n* = 35)**			**Older adults (*n* = 34)**		
	**Positive reappraisal (*n* = 18)**	**Detached reappraisal (*n* = 17)**	**Group comparisons (corrected *p*-value)**	**Positive reappraisal (*n* = 17)**	**Detached reappraisal (*n* = 17)**	**Group comparisons (corrected *p*-value)**
**Affective responses**						
Arousal rating (max. 9)	−0.3796 (1.2895)	−1.1476 (1.8199)	0.250	−0.2059 (1.5916)	−0.1.3235 (1.8648)	0.198
Valence rating (max. 9)	1.2130 (1.3368)	0.3529 (1.0815)	0.144	0.8039 (1.1935)	−0.7843 (1.3081)	0.035
**Expressive responses**						
Facial EMG Zygomaticus Major^a^	−0.0007 (0.0015)	0.0020 (0.0069)	0.060	0.0001 (0.0015)	−0.0000 (0.0006)	0.945
Facial EMG Corrugator Supercilii^a^	0.0000 (0.0012)	−0.0002 (0.0023)	0.766	−0.0001 (0.0005)	0.0002 (0.0005)	0.198
**Physiological responses**						
SCL’s magnitude (μS)	0.0077 (0.0647)	−0.0216 (0.0474)	0.766	−0.0154 (0.0231)	−0.0005 (0.0239)	0.198
HR (bpm)	0.8733 (4.7497)	−2.3197 (4.9224)	0.060	−1.0350 (3.5762)	−0.1307 (4.5720)	0.651
**Working memory**						
Mean ratio	0.0107 (1.3368)	0.0241 (0.1143)	0.766	0.0869 (0.0917)	0.0600 (0.0865)	0.221

Standard deviations are listed in parentheses. Group comparisons between differential scores were assessed by Mann–Whitney *U* test. ^*a*^Area under the curve, mV* sec. *p* values are corrected with the Hochberg procedure for controlling the family-wise error rate.

### Additional Control Analyses

We conducted non-parametric comparisons (Mann–Whitney) in each age group separately to verify whether the participants assigned to the detached and positive reappraisal conditions differed in their demographical, cognitive, and affective characteristics. Those revealed no statistically significant difference (all corrected *p*s > 0.05, cf. Supplementary Material and Tables 2, 3). In addition, we searched for correlations between the affective, expressive, physiological, and cognitive measurements and all the other demographical, affective, and cognitive variables for which we found statistically significant differences between age groups (i.e., music listening, hearing levels, Stroop Victoria IF, PANAS negative affect, and NEO-PI-R scores; cf. Table 1). These correlational analyses are aimed to identify potential confounding factors that may explain our results in another way than by age-related or type of reappraisal effects. For each set of correlations, a Bonferroni correction was used to account for the increased chance of a type I error associated with conducting multiple correlations. To this end, we adjusted the α level from 0.05 to 0.00006. No statistically significant correlations were found. For each age group, we searched for correlations between the affective ratings (i.e., valence and arousal), behavioral responses (i.e., EMG), physiological reactions (i.e., skin conductance level and heart beat), and cognitive costs (i.e., mean working span ratio) for the positive and detached reappraisal conditions, respectively (all the tables of correlations are presented as Supplementary Material and Tables 4–7). We also sought correlations between cognitive inhibition (i.e., Stroop interference score) and cognitive costs (i.e., mean working span ratio) for all participants whatever their age group. After correcting the α level from 0.05 to 0.005 using Bonferroni adjustment, no statistically significant correlation was found (*p* = 0.081).

## Discussion

In the current study, we investigated age-related differences in the use of cognitive reappraisal to regulate emotional responses to threatening musical excerpts. Wanting to reduce the effect of visual attentional deployment (a strategy that may be preferentially used by older adults to disengage from unpleasant events) as a confounding factor on cognitive reappraisal abilities, we used a musical source of emotion. This choice was motivated by the fact that auditory stimuli should prevent participants from reallocating perceptual attention away from the emotional source (except if they plugged their own ears). In a mixed-design framework, participants were instructed to simply listen to negative content of music (spontaneous response) and then to implement cognitive reappraisal to reduce the negative emotion elicited by the music. In the cognitive reappraisal condition, one half of the participants were asked to focus on positive aspects of music (positive reappraisal), and the other half was instructed to detach from what the music conveyed to them (detached reappraisal). Aiming for a full investigation of younger and older adults’ emotional responses, we scrutinized the effects of these instructions on affective ratings (Likert scales), facial expressions (facial EMG), physiological state (SCL, HR), and cognitive performances (working memory task) of the participants.

First, our findings give empirical evidence for an age-related effect in spontaneous response to threatening musical excerpts. In line with previous findings (Mather et al., 2004; Vieillard and Bigand, 2014), we found that, when getting older, a reduction of emotional processing for negative stimuli occurs. When instructed to simply listen to threatening music, older adults reported a more positive feeling associated with a smaller SCL in comparison with their younger counterparts. In accordance with previous findings, it appears that, even in the absence of instructed emotional regulation, older adults engage in spontaneous downregulation of their negative emotions (Mather et al., 2004). It is worth noting that younger and older adults judged threatening musical excerpts as moderately arousing. In that sense, our results extend a previous fMRI study providing evidence that, when presented with low arousing negative pictures, compared with younger adults, older adults show an increased spontaneous activity in the prefrontal areas that have been interpreted as suggesting that the regulation networks of older adults are chronically activated in response to low arousing negative stimuli (Dolcos et al., 2014). In the same vain, Samanez-Larkin and Carstensen (2011) postulate that emotion regulation in aging might be more automatic and less cognitively effortful due to chronically activated goals. Nevertheless, the notion of chronically activated goals remains somewhat unclear in the literature. Such anotion logically evokes the idea that the emotion regulation would be based on more automatic processes with aging. Intriguingly, the motivational perspective of SST pleads for the idea that the goal of voluntarily maintaining emotional well-being is chronically activated among older adults and postulates, at the same time, that the preference for positivity and well-being in older adults reflects controlled cognition. This is ambiguous about whether spontaneous emotion regulation is resource demanding or not. As outlined by Isaacowitz and Blanchard-Fields (2012), to date, the logical link postulated by the SST between the cognitive resources required by the positivity effect and the emotion-regulation activity has never received empirical support. This leaves open the question whether spontaneous emotion regulation in aging must be conceived as an automatic or more controlled process. In our study, older adults showed no significant gain in their working memory performances in the simply listening condition in comparison with their younger counterparts. Such a finding does not fully corroborate the hypothesis that the older adults’ spontaneous tendency to downregulate negative emotion depends on more automatic processes than in younger adults. This is not to say that spontaneous emotion regulation is synonymous with effortful activity. Previous findings indicating that working memory performances were disrupted by prior efforts at self-regulating some behaviors (i.e., controlling the focus of visual attention, inhibiting predominant writing tendencies, or exaggerating emotional expressions) has been interpreted as supporting the idea that prior effortful tasks may determine the following operation of executive processes (Schmeichel, 2007). Based on such results, the fact that, in our study, the experience of fear *per se* (i.e., simply listening condition) does not affect performances on the working memory task, whatever the age group, suggests that spontaneous emotion regulation is not cognitively demanding in younger or older adults. The fact remains that, even at an equivalent cognitive cost, affective and physiological outcomes show that older adults are more effective at spontaneously regulating their emotions than their younger counterparts.

When the participants were asked to implement a cognitive reappraisal of the threatening musical excerpts that consists of either positively reevaluating the music or detaching from it, no effects of emotion regulation were found on facial expressions (facial EMG) or physiological state (SCL, HR) regardless of the age group. Such findings indicate that the participants did not use a strategy of expressive suppression or enhancement to implement the emotion-regulation instruction. This suggests that the attempt to regulate emotions was not based on a control of behavioral expression, allowing us to think that the participants conformed to the instructions. The fact that neither the positive nor the detached reappraisal instruction elicited a significant increase of physiological arousal is also congruent with previous findings indicating that this kind of emotion regulation has a positive impact on the affective sphere because it is associated with a decreased negative emotion experience without any increase in physiological activation (Cutuli, 2014). In line with this, we observe that both younger and older adults report a reduced negative emotion (valence rating) in the case of positive reappraisal and a reduced arousal (arousal rating) in the case of detached reappraisal. At first glance, these results are consistent with previous results showing equal success in implementing positive and detached reappraisal for younger and older adults (Allard and Kensinger, 2017; Livingstone and Isaacowitz, 2018). However, this view is a little bit challenged by additional findings indicating that, when asked to apply detached reappraisal, the older adults of our sample reported a more negative emotional experience (valence rating) than when they were asked to simply listen to it although this is not the case in their younger counterparts. Even if they are congruent with the phenomenon of emotional dedifferentiation with age (Grühn and Scheibe, 2008; Vieillard et al., 2012), conflicting results observed in older adults are hardly fully explainable in the context of emotion-regulation skills. One could explain them speculating that explicit instructions of detached reappraisal invite adopting a distant/indifferent attitude toward the emotion stimuli directly affecting older adults’—who may show greater compliance with instructions—overt valence ratings (e.g., Henkel, 2014). With respect to the age-related effect on emotion-regulation abilities, current results cannot conclusively determine whether or not older adults use detached reappraisal less successfully than younger adults. This verifies discrepant findings in the literature and asks for further investigations.

As in the previous study conducted by Scheibe and Blanchard-Fields (2009), our results seem to indicate that consequences of cognitive reappraisal for cognitive functioning varies as a function of age group. But it also varies as a function of the type of cognitive reappraisal. When instructed to positively reappraise the threatening musical excerpts, older adults show a significant gain in their working memory performances in comparison with the simply listening condition as well as in comparison with their younger counterparts. A similar pattern of results has been observed for detached reappraisal except that no age-related effect was found. Such findings are in line with the general idea that older adults may be more effective at regulating emotion (e.g., Charles and Carstensen, 2010). Importantly, the current results demonstrate that implementing cognitive reappraisal (positive and detached) does not deplete the limited cognitive resources in older adults. This is not consistent with the claim that older adults would use cognitive resources to regulate their emotions (e.g., Kryla-Lighthall and Mather, 2009). Because our experiment was designed to prevent participants from reallocating auditory attention away from the musical emotions, current results provide further support for the distraction hypothesis of Scheibe and Blanchard-Fields (2009), according to which older adults would be more easily diverted by the working memory task, thus leading to a downregulation of negative emotion. Even if we do not find a statistically significant correlation between the cognitive effect of the implementation of the emotion-regulation activity and the inhibitory capacities of participants, the distraction hypothesis is consistent with the fact that, compared to their younger counterparts, older adults do display lower cognitive performances. Literature on aging revealed that, due to their limited cognitive resources, older adults tend to be more distracted by irrelevant information when they are instructed to perform a cognitive task. In the context of our experiment, the working memory task might have been considered as irrelevant information while implementing reappraisal instructions, at least at the first stage of processing, but might also quickly have worked as a *relevant* mean to divert older adults from their negative emotions. In other words, we suggest that, although instructed to intentionally reappraise negative emotions, older adults may prefer diverting from them the source of emotion rather than implementing a reappraisal strategy. This is in line with the observation that age is associated with an increased preference to choose distraction over reappraisal (Scheibe et al., 2015). Such a hypothesis also corroborates previous findings showing that, compared with those younger, older adults are better at reducing their attention to unpleasant musical stimuli (Vieillard and Bigand, 2014). In that respect, the current pattern of findings is in accordance with the DIT model (Labouvie-Vief, 2008) postulating that older adults become more efficient at downregulating emotion precisely because their cognitive resources are limited. The fact that, in our experiment, older adults judged threatening music as moderately intense is consistent with the idea that musical stimuli elicited enough emotional intensity to lead older adults to divert from it. Does this mean that the current findings do not match the SST model? If, as we suspect, older adults do not really implement a reappraisal strategy *per se* but rather prefer to decrease negative emotions through a distraction strategy, there is no strong empirical evidence to claim that positive and detached reappraisal are less cognitively costly in older than in younger adults. Thus, current findings do not strictly provide evidence against SST model.

Regarding the spontaneous responses to musical excerpts designed for our baseline condition, an unexpected age-related difference in affective and physiological consequences was found. When faced with musical excerpts, such as a tuning orchestra, the older adults reported feeling a more negative emotion and showed greater HR than their younger counterparts, suggesting that age groups were not equivalent in their emotional reaction to music. Although it remains difficult to explain why older adults were more reactive to orchestral sounds in a negative way, our above findings demonstrating that older adults spontaneously downregulate their negative emotion suggest that the older adults’ negative appraisal of orchestral sounds (i.e., tuning) did not correspond to a general age-related emotional bias likely to impact the measures of our study. However, the orchestral sounds and threatening musical excerpts were extracted from the classical and the modern repertoire, respectively, which could also explain the different results obtained. More generally, findings outline the difficulty of choosing musical excerpts fit for a baseline condition because musical stimuli of neutral emotional value do not really exist in the natural environment. The present study carries another limitation. Even if we choose to operationalize the cognitive reappraisal with two different instructions conveying either positive or detached reappraisal, our results seem to indicate that older participants may still prefer to use another way, such as distraction, to downregulate their negative emotions. This raises the question of whether and how participants follow verbal instructions in emotion-regulation studies. Further research is needed to shed a light on this key question.

Overall, the current study provides empirical evidence that older adults are better at downregulating their negative emotions. Our data might suggest that such ability is the result of older adults’ inclination to use a subsequent working memory task as a distractor, thus facilitating the disengagement from negative music. It may be argued that, because cognitive reappraisal is not required during the working memory task *per se*, cognitive resources required by cognitive reappraisal way not directly affect those in working memory task. As a consequence, current results could be explained in a different manner. For instance, it would be argued that the improved working memory performances in older adults reflect the more sustained mood states in this age group. Based on Dolcos et al. (2006) findings showing that the arousal level during the working memory task may explain the change in working memory performances, one could state that the improvement of working memory performances in older adults is the result of a higher level of arousal able to sustain their mood state. However, we found that the arousal ratings were lower in all participants whatever the age group across reappraisal conditions. This suggests that the improved working memory performances in older adults cannot be explained by a difference in the way the mood state is sustained for the duration of the working memory task. Regarding the question of whether the cognitive resources required by cognitive reappraisal do or do not directly affect those in working memory tasks, previous findings have persuasively demonstrated that prior efforts at executive control do have a significant effect on subsequent operations requiring executive processes (Schmeichel, 2007). Such results do not corroborate the idea that cognitive resources required in the emotional reappraisal task are completely independent of those involved in the working memory task. Based on the pattern of findings observed for older adults, no evidence was found to advocate that positive and detached reappraisal become less cognitively costly while getting old. However, our results indicate that attention processes play an important role in emotion-regulation success in the elderly. If we assume that, in older adults, a low resource–demanding regulation is the best guarantee of success, then the distraction strategy appears as a good candidate for them. This is consistent with the hypothesis, regularly demonstrated in the field of visual attention (e.g., Isaacowitz et al., 2006; Scheibe et al., 2015; Livingstone and Isaacowitz, 2018) that older adults would have pervasive preference to turn away from negative stimuli. It could be argued that the lack of a baseline measure of participant’s working memory capacity does not allow us to extract clear conclusions regarding our previous findings because we do not exactly know to what extent the working memory task may reduce cognitive gains or costs depending on the age group. However, the assessment of cognition, including working memory tasks (i.e., reverse counting and spelling, MMSE), provided a guarantee that older adults in our sample have a good level of executive functioning. The aim of the current experimental design was to investigate to what extent initial efforts at executive control required by a emotion-regulation task may affect subsequent efforts at implementing a working memory task. Previous findings observed in younger adults have shown that prior efforts at executive control do have a significant effect on subsequent operations requiring executive processes in this age group (Schmeichel, 2007). Regarding the literature on age-related effect on executive control, it is very unlikely that the magnitude of this effect would be reduced in older adults in comparison with their younger counterparts. At most, if we suppose that older adults in our sample are high functioning, they could have equivalent executive abilities as their younger counterparts. Yet our results indicated that the instruction to intentionally reappraise negative emotions results in a lesser cognitive cost for older adults in comparison with younger adults. This suggests that the potential variation (*a priori* down in older adults) in executive functioning performances across age groups could be an explaining factor for current results as follows: Older adults used the working memory task as a distractor to facilitate their disengagement from negative music. Even if they need further investigations, current findings are in line with the previous work of Scheibe and Blanchard-Fields (2009). Taken together with these previous findings, available empirical data indicate that the distraction strategy used by older adults to reduce their negative feelings applies at least to two types of negative emotions: disgust and fear. Further investigation is required to investigate other categories of feeling. Additional research efforts are also required to determine how situational factors encourage older adults to preferentially apply a specific emotion regulation over another.

## Data Availability Statement

The datasets generated for this study are available on request to the corresponding author.

## Ethics Statement

The studies involving human participants were reviewed and approved by the ANR, the Institutional Review Board that has funded the research program (ANR-11-EMCO-0003). The patients/participants provided their written informed consent to participate in this study.

## Author Contributions

SV designed the research and conducted the statistical analyzes. SV and EB validated the musical stimuli. SV, CP, and EB wrote the manuscript. All authors contributed to the article and approved the submitted version.

## Conflict of Interest

The authors declare that the research was conducted in the absence of any commercial or financial relationships that could be construed as a potential conflict of interest.
