# Supplementary material for: Positive and Detached Reappraisal of Threatening Music in Younger and Older Adults
Source: Front Hum Neurosci. 2020 Jun 25;14:216. doi: 10.3389/fnhum.2020.00216 (PMC7330061; doi:10.3389/fnhum.2020.00216)
Supplement: Supplementary file 1 [file Table_2.docx]

Supplementary Material

# Supplementary Tables

| *Table 1.* Description of musical excerpts' characteristics and their allocation under the different emotion regulation conditions | | | | | | | | | | | | | |
| --- | --- | --- | --- | --- | --- | --- | --- | --- | --- | --- | --- | --- | --- |
|  |  |  |  | | | | | | | | | | |
| **Battery Stimuli** | **Name of Musical Excerpts** |  | **Composer** |  | **Status** |  | **Positive Reappraisal^1^** |  | **Detached Reappraisal^1^** |  | **Positive Reappraisal^1^** |  | **Detached Reappraisal^1^** |
| Bigand et al. (2005) | Peaceful3_Traumerei, op. 15, no. 7 |  | Robert Schuman |  | Familiarization with ratings |  | Listening [01] |  | Listening [01] |  | Listening [01] |  | Listening [01] |
|  | Peaceful1_Sonata A for Harpsichord, K208 |  | Domenico Scarlatti |  | Debriefing |  | Listening [06] |  | Listening [06] |  | Listening [06] |  | Listening [06] |
|  | Peaceful2_Duetto for two flutes in F, Lamentabile |  | Wilhelm Friedemann Bach |  | Debriefing |  | Listening [06] |  | Listening [06] |  | Listening [06] |  | Listening [06] |
| Created for the study | Neutral1 |  | - |  | Baseline |  | Listening [02] |  | Listening [02] |  | Listening [02] |  | Listening [02] |
|  | Neutral2 |  | - |  | Baseline |  | Listening [02] |  | Listening [02] |  | Listening [02] |  | Listening [02] |
|  | Neutral3 |  | - |  | Baseline |  | Listening [02] |  | Listening [02] |  | Listening [02] |  | Listening [02] |
|  | Neutral5 |  | - |  | Baseline |  | Listening [02] |  | Listening [02] |  | Listening [02] |  | Listening [02] |
| Platel et al. (Unsubmitted) | Treathening2_Breaking The Key |  | Boardman, Chris |  | Training |  | Positive Reappraisal [04] |  | Detached Reappraisal [04] |  | Positive Reappraisal [04] |  | Detached Reappraisal [04] |
|  | Treathening3_The Story 1 |  | Danny Elfman |  | Training |  | Positive Reappraisal [04] |  | Detached Reappraisal [04] |  | Positive Reappraisal [04] |  | Detached Reappraisal [04] |
|  | Treathening5_The Cyclops |  | Bernard Herrmann |  | Test |  | Listening [03] |  | Listening [03] |  | Positive Reappraisal [05] |  | Detached Reappraisal [05] |
|  | Treathening6_Journey to the Center of the Earth |  | Bernard Herrmann |  | Test |  | Listening [03] |  | Listening [03] |  | Positive Reappraisal [05] |  | Detached Reappraisal [05] |
|  | Treathening7_Into The Woods_The Witch |  | Danny Elfman |  | Test |  | Listening [03] |  | Listening [03] |  | Positive Reappraisal [05] |  | Detached Reappraisal [05] |
|  | Treathening8_The Battle In The Mines; The Balrog |  | Leonard Rosenman |  | Test |  | Listening [03] |  | Listening [03] |  | Positive Reappraisal [05] |  | Detached Reappraisal [05] |
|  | Treathening9_Lilith Morphs |  | Boardman, Chris |  | Test |  | Listening [03] |  | Listening [03] |  | Positive Reappraisal [05] |  | Detached Reappraisal [05] |
|  | Treathening10_Mysterious Island - The Giant Crab |  | Bernard Herrmann |  | Test |  | Listening [03] |  | Listening [03] |  | Positive Reappraisal [05] |  | Detached Reappraisal [05] |
|  | Treathening11_The Church Battle |  | Danny Elfman |  | Test |  | Positive Reappraisal [05] |  | Detached Reappraisal [05] |  | Listening [03] |  | Listening [03] |
|  | Treathening12_Galen's Encounter |  | Alex North |  | Test |  | Positive Reappraisal [05] |  | Detached Reappraisal [05] |  | Listening [03] |  | Listening [03] |
|  | Treathening13_The Return |  | Bernard Herrmann |  | Test |  | Positive Reappraisal [05] |  | Detached Reappraisal [05] |  | Listening [03] |  | Listening [03] |
|  | Treathening14_The Church Battle 2 |  | Danny Elfman |  | Test |  | Positive Reappraisal [05] |  | Detached Reappraisal [05] |  | Listening [03] |  | Listening [03] |
|  | Treathening15_Elspeth At The Stake |  | Alex North |  | Test |  | Positive Reappraisal [05] |  | Detached Reappraisal [05] |  | Listening [03] |  | Listening [03] |
|  | Treathening16_The Dragon |  | Bernard Herrmann |  | Test |  | Positive Reappraisal [05] |  | Detached Reappraisal [05] |  | Listening [03] |  | Listening [03] |
|  |  |  |  |  |  |  |  |  |  |  |  |  |  |
| *Nota.* For each block [02], [03], [04], [05] the presentation order of musical stimuli was randomized ; ^1^ 25% of the participants. | | | | | | | | | | | | | |

**Table 2. Comparisons between old adults in the detached and the positive reappraisal conditions.**

|  | Detached Reappraisal (*n*=17) | | Positive Reappraisal (*n*=17) | | corrected *p* value |
| --- | --- | --- | --- | --- | --- |
|  | Mean | Standard deviation | Mean | Standard deviation |  |
| **Demographic Characteristics** |  |  |  |  |  |
| Age (year) | 68.53 | 5.09 | 63.88 | 3.22 | 0.13 |
| Education (year) | 13.82 | 2.19 | 13.94 | 1.89 | 0.93 |
| Music listening (hours per week) | 10.47 | 14.25 | 4.53 | 6.58 | 0.47 |
| Self reported health (max. 5) | 4.12 | 0.60 | 4.53 | 0.51 | 0.40 |
| **Hearing Level (dB)** |  |  |  |  |  |
| 500Hz | 15.88 | 5.79 | 15.15 | 5.41 | 0.86 |
| 1000Hz | 16.32 | 8.48 | 14.85 | 6.28 | 0.87 |
| 2000Hz | 23.09 | 13.91 | 14.71 | 10.68 | 0.19 |
| 4000Hz | 34.41 | 19.79 | 26.18 | 16.52 | 0.78 |
| 8000Hz | 50.29 | 21.50 | 44.56 | 21.00 | 0.86 |
| **Cognitive Scores** |  |  |  |  |  |
| Inhibition: Victoria Stroop (IF) | 2.05 | 0.32 | 2.03 | 0.34 | 0.86 |
| Working Memory: Digit Span (max. 30) | 9.71 | 1.36 | 10.59 | 2.35 | 0.80 |
| MMSE | 29.65 | 0.61 | 29.88 | 0.33 | 0.78 |
| **Affectives Scores** |  |  |  |  |  |
| PANAS Positive Affects (max. 50) | 34.94 | 3.78 | 34.41 | 5.61 | 0.87 |
| PANAS Negative Affects (max. 50) | 15.65 | 5.43 | 16.65 | 5.85 | 0.86 |
| BDI-II (max. 63) | 7.47 | 6.26 | 4.71 | 4.01 | 0.80 |
| STAI-Y Trait (max. 80) | 30.12 | 6.58 | 25.47 | 3.92 | 0.56 |
| STAI-Y State (max. 80) | 35.47 | 5.66 | 34.71 | 7.41 | 0.86 |
| **Emotion Regulation Questionnaire Scores** |  |  |  |  |  |
| ERQ Suppression Score (max. 28) | 14.24 | 5.23 | 13.12 | 4.57 | 0.86 |
| ERQ Regulation Score (max. 42) | 30.24 | 5.26 | 28.82 | 6.86 | 0.86 |
| **NEO PIR Scores** |  |  |  |  |  |
| Neuroticism (max. 192) | 85.59 | 18.58 | 80.65 | 13.61 | 0.86 |
| Extraversion (max. 192) | 104.00 | 11.12 | 102.06 | 13.34 | 0.86 |
| Openess to experience (max. 192) | 115.82 | 14.79 | 119.24 | 9.62 | 0.80 |
| Consciousness (max. 192) | 130.76 | 12.21 | 131.29 | 16.69 | 0.86 |
| Agreeableness (max. 192) | 121.94 | 10.30 | 120.88 | 15.59 | 0.91 |

*p* values are corrected with the Hochberg procedure for controlling the family-wise error rate

**Table 3. Comparisons between young adults in the detached and the positive reappraisal conditions.**

|  | Detached Reappraisal (*n*=17) | | Positive Reappraisal (*n*=18) | | corrected *p* value |
| --- | --- | --- | --- | --- | --- |
|  | Mean | Standard deviation | Mean | Standard deviation |  |
| **Demographic Characteristics** |  |  |  |  |  |
| Age (year) | 20.59 | 2.09 | 20.78 | 2.39 | 0.99 |
| Education (year) | 13.24 | 1.60 | 13.06 | 1.55 | 0.99 |
| Music listening (hours per week) | 8.06 | 6.48 | 13.72 | 13.63 | 0.99 |
| Self reported health (max. 5) | 4.24 | 0.75 | 4.61 | 0.50 |  |
| **Hearing Level (dB)** |  |  |  |  | 0.99 |
| 500Hz | 8.97 | 8.20 | 7.92 | 3.24 | 0.99 |
| 1000Hz | 7.79 | 10.07 | 5.97 | 4.71 | 0.99 |
| 2000Hz | 3.53 | 7.35 | 2.64 | 6.44 | 0.99 |
| 4000Hz | 0.59 | 5.90 | 0.56 | 8.85 | 0.99 |
| 8000Hz | 5.29 | 8.00 | 8.33 | 12.86 | 0.99 |
| **Cognitive Scores** |  |  |  |  |  |
| Inhibition: Victoria Stroop (IF) | 1.79 | 0.36 | 1.74 | 0.37 | 0.99 |
| Working Memory: Digit Span (max. 30) | 11.18 | 2.27 | 11.11 | 3.38 | 0.99 |
| **Affectives Scores** |  |  |  |  |  |
| PANAS Positive Affects (max. 50) | 31.65 | 6.33 | 33.94 | 5.36 | 0.99 |
| PANAS Negative Affects (max. 50) | 19.18 | 8.80 | 18.78 | 4.81 | 0.99 |
| BDI-II (max. 63) | 6.71 | 4.82 | 8.61 | 5.62 | 0.99 |
| STAI-Y Trait (max. 80) | 27.35 | 4.27 | 31.00 | 5.42 | 0.99 |
| STAI-Y State (max. 80) | 38.71 | 6.74 | 37.61 | 6.49 | 0.99 |
| **Emotion Regulation Questionnaire Scores** |  |  |  |  |  |
| ERQ Suppression Score (max. 28) | 17.53 | 4.94 | 14.22 | 5.50 | 0.99 |
| ERQ Regulation Score (max. 42) | 26.82 | 7.18 | 28.61 | 4.22 | 0.99 |
| **NEO PIR Scores** |  |  |  |  |  |
| Neuroticism (max. 192) | 92.18 | 25.08 | 87.61 | 19.86 | 0.99 |
| Extraversion (max. 192) | 112.00 | 17.05 | 116.28 | 17.06 | 0.99 |
| Openess to experience (max. 192) | 121.94 | 16.80 | 124.39 | 20.30 | 0.99 |
| Consciousness (max. 192) | 118.76 | 26.73 | 124.17 | 22.13 | 0.99 |
| Agreeableness (max. 192) | 111.06 | 20.77 | 118.89 | 21.97 | 0.99 |

*p* values are corrected with the Hochberg procedure for controlling the family-wise error rate

**Table 4. Correlations between the experimental measures and the age-related demographical, cognitive and emotional characteristics in the old-adults group using positive reappraisal (*n*=17).**

|  | Arousal rating | Valence rating | Facial EMG Zygomaticus Major (Area under the curve, mV*sec) | Facial EMG Corrugator Supercilii (Area under the curve, mV*sec) | SCL' s Magnitude (µS) | HR (bpm) | Working Memory Task's Performances (mean ratio) |
| --- | --- | --- | --- | --- | --- | --- | --- |
| Music listening per week | 0.63 | 0.35 | 0.05 | -0.17 | 0.50 | 0.28 | 0.08 |
| STROOP IF | 0.15 | 0.15 | -0.72 | 0.45 | -0.04 | -0.04 | -0.23 |
| PANAS Negative Affects | -0.65 | -0.35 | 0.19 | 0.16 | -0.35 | -0.19 | 0.01 |
| NEO PI-R Neuroticism | -0.33 | -0.16 | 0.15 | 0.22 | 0.21 | 0.39 | -0.28 |
| NEO PI-R Extraversion | 0.33 | 0.70 | -0.36 | -0.22 | 0.03 | 0.24 | 0.11 |
| NEO PI-R Openess to experience | -0.23 | -0.26 | 0.25 | 0.23 | -0.31 | 0.34 | -0.16 |
| NEO PI-R Agreeableness | -0.01 | 0.19 | -0.08 | -0.36 | 0.00 | 0.45 | 0.24 |
| NEO PI-R Consciousness | 0.02 | 0.11 | 0.07 | -0.09 | -0.35 | 0.12 | 0.59 |
| Hearing Level (dB) 500Hz | 0.04 | -0.29 | -0.35 | 0.35 | 0.50 | -0.20 | -0.53 |
| Hearing Level (dB) 1000Hz | 0.06 | -0.32 | -0.29 | 0.27 | 0.60 | -0.13 | -0.38 |
| Hearing Level (dB) 2000Hz | 0.31 | -0.01 | -0.67 | 0.54 | 0.11 | -0.29 | -0.28 |
| Hearing Level (dB) 4000Hz | 0.26 | 0.01 | -0.38 | 0.21 | 0.11 | -0.36 | 0.28 |
| Hearing Level (dB) 8000Hz | 0.24 | 0.02 | -0.38 | 0.09 | 0.18 | -0.34 | 0.28 |

**Table 5. Correlations between the experimental measures and the age-related demographical cognitive and emotional characteristics in the young-adults group using positive reappraisal (*n*=18).**

|  | Arousal rating | Valence rating | Facial EMG Zygomaticus Major (Area under the curve, mV*sec) | Facial EMG Corrugator Supercilii (Area under the curve, mV*sec) | SCL' s Magnitude (µS) | HR (bpm) | Working Memory Task's Performances (mean ratio) |
| --- | --- | --- | --- | --- | --- | --- | --- |
| Music listening per week | 0.11 | 0.23 | -0.32 | -0.21 | 0.02 | 0.16 | -0.29 |
| STROOP IF | -0.19 | -0.35 | -0.07 | -0.15 | 0.09 | 0.08 | 0.05 |
| PANAS Negative Affects | 0.18 | -0.22 | 0.00 | 0.39 | -0.28 | -0.35 | 0.28 |
| NEO PI-R Neuroticism | -0.11 | 0.06 | 0.13 | -0.01 | -0.27 | -0.50 | 0.00 |
| NEO PI-R Extraversion | 0.21 | -0.20 | 0.07 | -0.33 | 0.11 | 0.35 | -0.21 |
| NEO PI-R Openess to experience | -0.23 | 0.37 | 0.16 | -0.24 | -0.28 | 0.34 | -0.17 |
| NEO PI-R Agreeableness | 0.14 | 0.18 | 0.26 | 0.08 | 0.29 | 0.57 | 0.09 |
| NEO PI-R Consciousness | -0.04 | -0.18 | -0.34 | 0.00 | 0.21 | 0.17 | -0.07 |
| Hearing Level (dB) 500Hz | 0.01 | -0.40 | -0.22 | -0.01 | -0.18 | -0.39 | 0.59 |
| Hearing Level (dB) 1000Hz | 0.16 | -0.26 | -0.08 | 0.05 | -0.16 | -0.22 | 0.40 |
| Hearing Level (dB) 2000Hz | 0.21 | -0.25 | -0.17 | 0.22 | -0.03 | -0.41 | 0.32 |
| Hearing Level (dB) 4000Hz | 0.14 | -0.24 | -0.36 | -0.01 | -0.04 | -0.31 | 0.42 |
| Hearing Level (dB) 8000Hz | 0.15 | -0.16 | 0.03 | 0.09 | -0.19 | -0.14 | 0.07 |

**Table 6. Correlations between the experimental measures and the age-related demographical, cognitive and emotional characteristics in the old-adults group using detached reappraisal (*n*=17).**

|  | Arousal rating | Valence rating | Facial EMG Zygomaticus Major (Area under the curve, mV*sec) | Facial EMG Corrugator Supercilii (Area under the curve, mV*sec) | SCL' s Magnitude (µS) | HR (bpm) | Working Memory Task's Performances (mean ratio) |
| --- | --- | --- | --- | --- | --- | --- | --- |
| Music listening per week | -0.17 | -0.25 | -0.08 | 0.23 | 0.20 | 0.23 | 0.00 |
| STROOP IF | 0.07 | 0.07 | 0.32 | 0.06 | -0.65 | 0.07 | -0.34 |
| PANAS Negative Affects | -0.12 | 0.15 | -0.07 | 0.36 | -0.40 | 0.31 | 0.05 |
| NEO PI-R Neuroticism | 0.10 | 0.15 | -0.15 | -0.27 | 0.25 | 0.08 | -0.25 |
| NEO PI-R Extraversion | -0.20 | -0.08 | -0.15 | 0.14 | 0.18 | 0.03 | 0.23 |
| NEO PI-R Openess to experience | -0.28 | -0.13 | -0.30 | 0.41 | -0.04 | 0.00 | 0.23 |
| NEO PI-R Agreeableness | 0.00 | -0.29 | -0.26 | -0.03 | 0.12 | -0.32 | 0.16 |
| NEO PI-R Consciousness | 0.03 | 0.21 | -0.12 | -0.39 | -0.24 | 0.19 | 0.25 |
| Hearing Level (dB) 500Hz | 0.12 | -0.09 | -0.16 | 0.18 | -0.38 | 0.01 | -0.26 |
| Hearing Level (dB) 1000Hz | -0.05 | -0.05 | 0.07 | 0.50 | -0.24 | -0.01 | -0.06 |
| Hearing Level (dB) 2000Hz | -0.12 | -0.16 | 0.07 | 0.21 | 0.00 | -0.20 | -0.15 |
| Hearing Level (dB) 4000Hz | 0.15 | -0.12 | -0.04 | 0.12 | -0.26 | -0.22 | -0.27 |
| Hearing Level (dB) 8000Hz | 0.05 | -0.42 | -0.04 | 0.08 | -0.34 | -0.23 | -0.49 |

**Table 7. Correlations between the experimental measures and the age-related demographical, cognitive and emotional characteristics in the young-adults group using detached reappraisal (*n*=17).**

|  | Arousal rating | Valence rating | Facial EMG Zygomaticus Major (Area under the curve, mV*sec) | Facial EMG Corrugator Supercilii (Area under the curve, mV*sec) | SCL' s Magnitude (µS) | HR (bpm) | Working Memory Task's Performances (mean ratio) |
| --- | --- | --- | --- | --- | --- | --- | --- |
| Music listening per week | 0.38 | 0.43 | 0.18 | 0.18 | 0.20 | 0.03 | 0.06 |
| STROOP IF | -0.05 | -0.20 | -0.04 | -0.08 | -0.29 | 0.15 | 0.21 |
| PANAS Negative Affects | 0.17 | -0.24 | 0.54 | 0.05 | -0.16 | 0.14 | -0.20 |
| NEO PI-R Neuroticism | -0.03 | 0.26 | 0.23 | -0.16 | 0.18 | -0.07 | 0.19 |
| NEO PI-R Extraversion | -0.17 | -0.06 | -0.09 | -0.17 | 0.15 | -0.30 | -0.10 |
| NEO PI-R Openess to experience | -0.18 | -0.01 | 0.42 | 0.24 | -0.20 | 0.44 | 0.19 |
| NEO PI-R Agreeableness | 0.31 | -0.22 | 0.22 | -0.19 | -0.09 | -0.09 | -0.33 |
| NEO PI-R Consciousness | -0.18 | -0.07 | -0.02 | -0.09 | 0.08 | -0.03 | -0.19 |
| Hearing Level (dB) 500Hz | -0.23 | -0.09 | -0.22 | -0.28 | 0.34 | -0.09 | -0.24 |
| Hearing Level (dB) 1000Hz | -0.04 | 0.03 | 0.02 | -0.19 | 0.13 | -0.01 | -0.29 |
| Hearing Level (dB) 2000Hz | 0.43 | 0.42 | 0.07 | -0.11 | 0.12 | -0.01 | 0.12 |
| Hearing Level (dB) 4000Hz | 0.01 | -0.24 | 0.25 | 0.36 | 0.03 | -0.04 | -0.21 |
| Hearing Level (dB) 8000Hz | 0.17 | -0.11 | -0.35 | -0.07 | 0.08 | -0.35 | -0.28 |
